# Supplementary material for: Intrinsic Gain Modulation and Adaptive Neural Coding
Source: PLoS Comput Biol. 2008 Jul 18;4(7):e1000119. doi: 10.1371/journal.pcbi.1000119 (PMC2440820; doi:10.1371/journal.pcbi.1000119)
Supplement: Text S1. — Firing Rate of the LIF Model with Noisy Stimuli. (0.09 MB DOC) [file pcbi.1000119.s001.doc]

# Text S1. Supporting Information

# *Intrinsic gain modulation and adaptive neural coding*

Sungho Hong, Brian Nils Lundstrom and Adrienne L. Fairhall

# Firing rate of the LIF model with noisy stimuli

There are known analytic formulas for the firing rate of the simple neuron models given Gaussian noise stimuli such as the LIF [1-3] and QIF [4,5]. Fourcaud-Trocmé et al. [6] obtained a formula for a large class of models including the LIF and QIF. Here we use them to discuss two aspects of the LIF model.

First, we show that the firing rate always increases as variance increases. The analytic form of the firing rate is [1-3]

$$f(I_{0},\sigma^{2})=\left(\tau\int_{\frac{CV_{r}-I_{0}\tau}{\sigma\tau}}^{\frac{C\theta-I_{0}\tau}{\sigma\tau}} \sqrt{\pi}e^{x^{2}}\left(1+\mathrm{erf}(x)\right)dx\right)^{-1},$$

where $\tau=1/g_{L}$. For convenience, we define $J(x)\equiv e^{x^{2}}\left(1+\mathrm{erf}(x)\right)$. The firing rate change with variance is given by

$$\begin{matrix} \frac{1}{f^{2}}\frac{\partial f}{\partial\sigma^{2}}=\frac{\tau\sqrt{\pi}}{2}\left(\frac{C\theta-I_{0}\tau}{\sigma^{3}\tau}\right)\cdot J\left(\frac{C\theta-I_{0}\tau}{\sigma\tau}\right)-\frac{\tau\sqrt{\pi} }{2}\left(\frac{CV_{r}-I_{0}\tau}{\sigma^{3}\tau}\right)\cdot J\left(\frac{CV_{r}-I_{0}\tau}{\sigma\tau}\right) \\ =\left.\frac{\tau\sqrt{\pi}}{2\sigma^{2}}xJ(x)\right|_{\frac{CV_{r}-I_{0}\tau}{\sigma^{3}\tau}}^{\frac{C\theta-I_{0}\tau}{\sigma\tau}}. \end{matrix}$$

Now $xJ(x)$ is an increasing function of $x$ whose minimum is $\lim_{x\to-\infty} xJ(x)=-\frac{1}{\sqrt{\pi}}$. Therefore, $\frac{\partial f}{\partial\sigma^{2}}$ is always positive and the firing rate also always increases with variance.

However, with a larger variance, the change in the firing rate becomes smaller and the firing rate approaches an asymptotic limit. In the limit $\sigma^{2}\to\infty$and $I_{0}/\sigma\to\text{finite}$, Eq. vanishes in the leading order. The next leading order survives as

$f=\left[C\left(\frac{\theta-V_{r}}{\sigma}\right)J\left(-I_{0}/\sigma\right)\right]^{-1}$.

Note that Eq. is factorized in a similar way to Eq. (8) in the paper. Therefore, the rescaled relative gain is

$$\frac{\sigma}{f}\frac{\partial f}{\partial I_{0}}=\frac{J'(-I_{0}/\sigma)}{J(-I_{0}/\sigma)},$$

which is a function only of $I_{0}/\sigma$ as we have seen in Fig. 3C. Note that the firing rate in this limit has a form of a function of$I_{0}/\sigma$ multiplied by a factor which only depends on the variance, and this makes the rescaled relative gain only a function of $I_{0}/\sigma$.

In the QIF case, the firing rate is [4,5]

$$f=\frac{\sigma^{3/2}}{\pi^{1/2}}\left(\int_{-\infty}^{\infty} d\zeta\exp\left[-\frac{1}{\sigma^{1/2}}\left((I_{0}/\sigma)^{2}\zeta^{2}+\zeta^{6}/48\right)\right]\right)^{-1}$$

where we have taken $C=1$ and $L(V)=V^{2}$ for simplicity. Here, we do not have a simple factorization as Eq. in the $\sigma\to\infty$ limit. Therefore, the rescaling is not directly related to its dynamics, but is rather phenomenological and approximate.

# References

1. Siegert AJF (1951) On the first passage time probability function. Phys Rev 81: 617-623.

2. Ricciardi LM (1997) Diffusion processes and related topics in biology. Berlin: Springer-Verlag.

3. Amit DJ, Tsodyks MV (1991) Quantitative study of attractor neural network retrieving at low spike rates: I. Substrate-spikes, rates and neuronal gain. Network 2: 259-273.

4. Lindner B, Longtin A, Bulsara A (2003) Analytic expressions for rate and CV of a type I neuron driven by white gaussian noise. Neural Comput 15: 1760-1787.

5. Brunel N, Latham PE (2003) Firing rate of the noisy quadratic integrate-and-fire neuron. Neural Comput 15: 2281-2306.

6. Fourcaud-Trocmé N, Hansel D, van Vreeswijk C, Brunel N (2003) How spike generation mechanisms determine the neuronal response to fluctuating inputs. J Neurosci 23: 11628-11640.
